# CLIPdb: a CLIP-seq database for protein-RNA interactions

**DOI:** 10.1186/s12864-015-1273-2

**Published:** 2015-02-05

**Authors:** Yu-Cheng T Yang, Chao Di, Boqin Hu, Meifeng Zhou, Yifang Liu, Nanxi Song, Yang Li, Jumpei Umetsu, Zhi John Lu

**Affiliations:** MOE Key Laboratory of Bioinformatics, Center for Synthetic and Systems Biology and Center for Plant Biology, School of Life Sciences, Tsinghua University, Beijing, 100084 China; Department of Biological Information, Tokyo Institute of Technology, Tokyo, 152-8850 Japan

**Keywords:** CLIP-seq, RNA-binding protein, RNA, Regulatory networks, Data integration

## Abstract

**Background:**

RNA-binding proteins (RBPs) play essential roles in gene expression regulation through their interactions with RNA transcripts, including coding, canonical non-coding and long non-coding RNAs. Large amounts of crosslinking immunoprecipitation (CLIP)-seq data (including HITS-CLIP, PAR-CLIP, and iCLIP) have been recently produced to reveal transcriptome-wide binding sites of RBPs at the single-nucleotide level.

**Description:**

Here, we constructed a database, CLIPdb, to describe RBP-RNA interactions based on 395 publicly available CLIP-seq data sets for 111 RBPs from four organisms: human, mouse, worm and yeast. We consistently annotated the CLIP-seq data sets and RBPs, and developed a user-friendly interface for rapid navigation of the CLIP-seq data. We applied a unified computational method to identify transcriptome-wide binding sites, making the binding sites directly comparable and the data available for integration across different CLIP-seq studies. The high-resolution binding sites of the RBPs can be visualized on the whole-genome scale using a browser. In addition, users can browse and download the identified binding sites of all profiled RBPs by querying genes of interest, including both protein coding genes and non-coding RNAs.

**Conclusion:**

Manually curated metadata and uniformly identified binding sites of publicly available CLIP-seq data sets will be a foundation for further integrative and comparative analyses. With maintained up-to-date data sets and improved functionality, CLIPdb (http://clipdb.ncrnalab.org) will be a valuable resource for improving the understanding of post-transcriptional regulatory networks.

**Electronic supplementary material:**

The online version of this article (doi:10.1186/s12864-015-1273-2) contains supplementary material, which is available to authorized users.

## Background

RNA binding proteins (RBPs) play essential roles in the co-transcriptional and post-transcriptional regulation of gene expression [1-3]. In recent years, much progress has been made in the field of ribonomics, which uses high-throughput technologies to investigate the interactions between RBPs and their target RNAs, including coding and non-coding RNAs, in a quantitative and high-resolution manner. With the development of next-generation sequencing technologies, crosslinking immunoprecipitation (CLIP)-seq technology [4], which includes high-throughput sequencing (HITS)-CLIP [5], photoactivatable ribonucleoside-enhanced (PAR)-CLIP [6] and individual-nucleotide resolution crosslinking immunoprecipitation (iCLIP) [7], has become a powerful tool to study the transcriptome-wide *in vivo* binding sites of RBPs at the single-nucleotide level. CLIP-seq provides higher resolution than previous technologies (e.g., RNA Immunoprecipitation) for identifying protein binding sites on RNAs.

Approximately 400 CLIP-seq samples from about 80 publications are publicly available from various model organisms. However, the many inconsistencies existing in the metadata annotations for these samples, such as RBP names, tissue types, cell types and disease states, create challenges for public data sharing and reuse. Notably, the analyses of most CLIP-seq studies emphasize only the binding signatures of the RBPs on mRNAs, whereas the binding information is often ignored for long non-coding RNAs (lncRNAs) in raw CLIP-seq data. With the emerging important roles of lncRNAs [8-10], a database without metadata annotation inconsistencies will help researchers to examine the post-transcriptional regulatory mechanisms of lncRNAs.

Several resources, such as CLIPZ [11], doRiNA [12], starBase v2.0 [13] and AURA 2 [14], have provided annotations for some CLIP-seq studies and locations of RBP binding sites. However, most of these resources focus only on a small number of the existing CLIP-seq experiments. For instance, doRiNA contains data for 27 human RBPs, AURA 2 provides data for 47 RBPs, and starBase v2.0 provides data for 49 RBPs. Other databases, such as NPInter v2.0, which focuses on ncRNA-centric interactions [15], integrate various resources (e.g., starBase). Therefore, although these integrative databases cover more RBPs and species and have a broader perspective on various interactions, they usually include fewer CLIP-seq data sets and provide few high-resolution binding sites on RNAs. In addition, the heterogeneous results obtained using different resources hinder further comparison and integration of the data sets across different cell lines, tissues and developmental stages. Thus, a more complete and better-curated database of CLIP-seq studies is needed.

**Figure 1:**
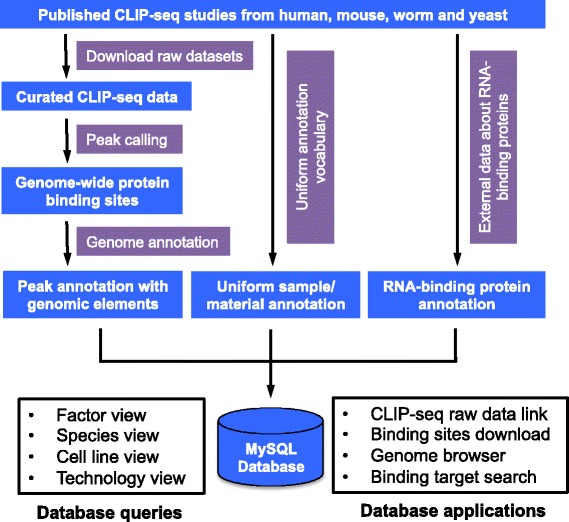
**CLIPdb data flow and framework.** Binding sites are called using analytical procedures identical to those for published CLIP-seq data sets. Additional information about the CLIP-seq data sets and RBPs are collected from external databases. All metadata are organized for convenient queries and applications. The details are described in the Construction and Content section.

Here, we describe CLIPdb (Figure 1), a database of various high-resolution binding sites for RBPs that we constructed using published CLIP-seq data. It contains manually curated annotations from all CLIP-seq studies across various model organisms. The database provides a user-friendly web interface, allowing researchers to rapidly find RBPs and CLIP-seq data of interest. CLIPdb also provides genome-wide binding sites for each data set, which are identified using a unified analysis procedure. The high-resolution binding site data from a large number of RBPs will benefit investigations on the coordination and competition of RBP binding, which has not been extensively studied. CLIPdb will also be of great value to researchers studying the post-transcriptional mechanisms regulating RNA splicing, stability and translation.

## Construction and content

### Collection and annotation of published CLIP-seq data sets

We curated published CLIP-seq data sets for four model organisms, *Homo sapiens*, *Mus musculus*, *Caenorhabditis elegans* and *Saccharomyces cerevisiae*, from public data repositories, including Gene Expression Omnibus (GEO), European Nucleotide Archive (ENA) and authors’ websites. In total, CLIPdb collected 395 CLIP-seq samples. We consistently annotated the samples with the following categories: RBP factor, species, tissue type, material (i.e., cell line, tissue and cell), cell type, cell line ID, disease state, treatment, assay method, data accession and reference.

### Pre-processing of CLIP-seq data sets

Different CLIP libraries may have very different complexities and signal-to-noise ratios depending on many experimental factors. We used multi-step and uniform procedures to pre-process the collected raw CLIP-seq data. First, we combined sequencing runs from the same sample. Then, we trimmed the adaptor and barcode sequences from the raw reads using the FASTX-Toolkit package [16]. We set a stringent read quality for each data set, retaining only those reads with a quality score above 20 in 80% of their nucleotides, and we also restricted the read length to 13 nucleotides (nt) after adapter trimming. Finally, we collapsed identical reads to minimize PCR duplicates. Detailed information for the CLIP-seq data sets we used and processed is shown in Additional file 1.

### Identification of transcriptome-wide binding sites

After pre-processing, retained reads from all samples were aligned to their respective genomes (human, hg19; mouse, mm10; yeast, R64-1-1; worm, ws220) using Bowtie-1.0.0 [17]. We retained only those reads with unique mapping locations by setting the Bowtie parameter as “-m 1 --best --strata.” To identify RBP binding sites, we applied Piranha (v1.2.0) [18] to all the samples, using the following parameters: −b 20 -d ZeroTruncatedNegativeBinomial -p 0.01 or 0.001. Piranha is a statistically robust and flexible computational method for identifying binding sites from CLIP-seq data, and is applicable to all variations of the CLIP-seq technologies. Piranha allows for the direct comparison of binding sites across different cell lines or tissues [18]. Notably, the binding sites obtained from Piranha software are not strand-specific because the current Piranha version does not provide strandness information. In addition, for some CLIP-seq samples (14 data sets), the raw data could not be easily accessed or were of such low quality, according to our results, that we did not provide their binding sites (see Additional file 1).

In addition to the peaks indiscriminately called by Piranha, we also provided the binding peaks called by specialized tools for different CLIP-seq technologies. For PAR-CLIP, we used PARalyzer to identify the T-C transitions [19]. For HITS-CLIP, we used CIMS to identify the mutation sites generated by UV crosslinking [20,21]. For iCLIP, we used CIMS/CITS (included in CIMS v1.0.3) to discriminate the truncation sties [22]. The default parameters were used when applying these specialized tools to identify binding sites.

### Annotation for binding sites of RBPs

We first used Gencode human (version 19), Gencode mouse (version M2), SGD (version R64-1-1) and WormBase (version ws220) to annotate the binding sites in the human, mouse, yeast and worm, respectively. In addition, for miRNAs, we used the latest version of annotations from miRBase [23]. For transposable elements (TEs) in the human and mouse, we downloaded the annotation from UCSC Genome Browser. For lncRNAs of the worm, we used the annotations from Nam et al., 2012 [24]. Binding sites with a significance cutoff of 0.01 by Piranha were overlapped with the genomic annotations of the coding sequences (CDS), 5′ untranslated regions (UTRs), 3′ UTRs, TEs, introns, lncRNAs, microRNA primary transcripts, canonical ncRNAs (snRNA, snoRNA, rRNA, tRNA, 7SK_RNA, Y_RNA), intergenic regions and other locations. Here an “intergenic region” denoted a constant distance from any genic region (coding genes, ncRNAs, TEs and pseudogenes), fixed at 2,000 nt for the human and mouse, and 500 nt for the worm and yeast. When 50% of the nucleotides in the binding sites overlapped with the genomic annotations, they were correspondingly annotated. We annotated the binding sites for the RBPs based on the following priority scheme: 3′ UTR, CDS, 5′ UTR, intron, microRNA primary transcript, canonical ncRNA, lncRNA, TE, pseudogene, intergenic region and others.

### Annotation of RBPs

The RBPs corresponding to these CLIP-seq data were further annotated with detailed information that was manually curated from other databases, such as NCBI [25], CISBP-RNA [26] and RBPDB [27]. Each RBP contained the following terms: the gene names for the RBP factors, species, Ensembl ID, synonyms and RNA binding domain. The RNA binding motifs of some RBPs were depicted in greater detail, including the RNA recognition motif sequence, position weight matrix of the motif, assay method and source literature.

In addition, all RBPs were classified into three major functional groups according to their molecular functional annotations provided in NCBI: (i) splicing factors, (ii) 3′ UTR/poly(A) binding factors and (iii) microRNA binding factors. The remaining RBPs were not grouped. We annotated the functions of the RBPs based on the following priority scheme: splicing factors, 3′ UTR/poly(A) binding factors, microRNA binding factors, and others (Additional file 2).

## Utility and discussion

### Basic characteristics of CLIPdb

**Figure 2:**
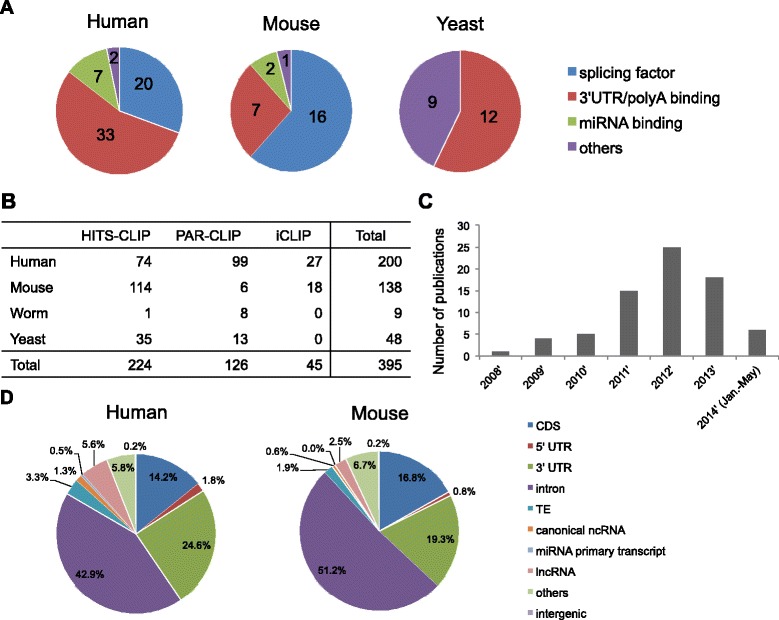
**Basic characteristics of CLIPdb. (A)** Distributions of functional groups of RBPs in the human, mouse and yeast. **(B)** Summary of CLIP-seq data sets. **(C)** Number of published CLIP-seq papers. **(D)** Genomic distributions of RBP binding sites in the human and mouse identified using Piranha.

CLIPdb collected 111 RBPs that were studied using CLIP-seq technology in four species. The data for humans, mice and yeast are summarized in Figure 2A. To date, only two RBPs have been collected for worms (Additional file 2). Splicing factors and 3′ UTR/poly(A) binding factors have been frequently studied, indicating their essential roles in RBP-based regulation. In addition, miRNA binding factors, especially AGO proteins, have also been well studied by CLIP-seq technologies, which is especially helpful for characterizing genome-wide microRNA binding signatures. Notably, in addition to CLIP-seq studies investigating one RBP at a time, global RBP-RNA interactions have been profiled in humans [28] and yeast [29], which may provide novel insights into co-binding patterns of RBPs. In total, there are 395 CLIP-seq samples, including 224 HITS-CLIP samples, 126 PAR-CLIP samples and 45 iCLIP samples (Figure 2B). Among these CLIP-seq data sets, approximately 50% (200) and 35% (138) were profiled in the human and mouse, respectively.

From statistical analyses, we observed that the total number of CLIP-seq studies published increased dramatically after 2008 and exhibited a gradual upward trend thereafter (Figure 2C). These results suggest that the investigation of protein-RNA interactions and CLIP-seq technology is becoming increasingly popular and important within the scientific community. We anticipate that more CLIP-seq studies in various cell types and tissues will be published in the future.

We analyzed the distribution of the genomic elements for the RBP binding sites from each sample in the four species (summarized in Figure 2D and Additional file 3: Figures S1 and S2). After pooling the binding sites in all RBPs for each species, we found that the human and mouse exhibited similar genomic elements, suggesting that functional binding patterns are conserved between mammals. The 3′ UTRs have more binding sites than the 5′ UTRs across all four species. Interestingly, this trend was most obvious for humans and mice, suggesting that 3′ UTR binding may have important regulatory functions in higher species. In addition to protein-coding regions, approximately 2-6% of all binding sites were located in mammalian lncRNAs, indicating that RBP binding may regulate the cellular functions of lncRNAs.

### Querying CLIPdb

The “binding sites navigation” module of CLIPdb contains four view tabs. The first tab is a “factor” view. Users select the RBPs of interest and obtain detailed information from the studies displayed in the data matrix. Users are also provided links to data sources and the appropriate literature. The RBP annotation matrices are available only under this factor view tab. The second tab is a “species” view in which users search CLIP-seq data sets in specific species. The third tab is a “cell line” view for searching the RBPs that have been profiled in specific cell lines, such as HeLa and HEK293 cells. The fourth tab is a “technology” view for users to search CLIP-seq studies according to the CLIP technologies used, including HITS-CLIP, iCLIP, PAR-CLIP and gPAR-CLIP.

### Downloading and visualizing binding sites

**Figure 3:**
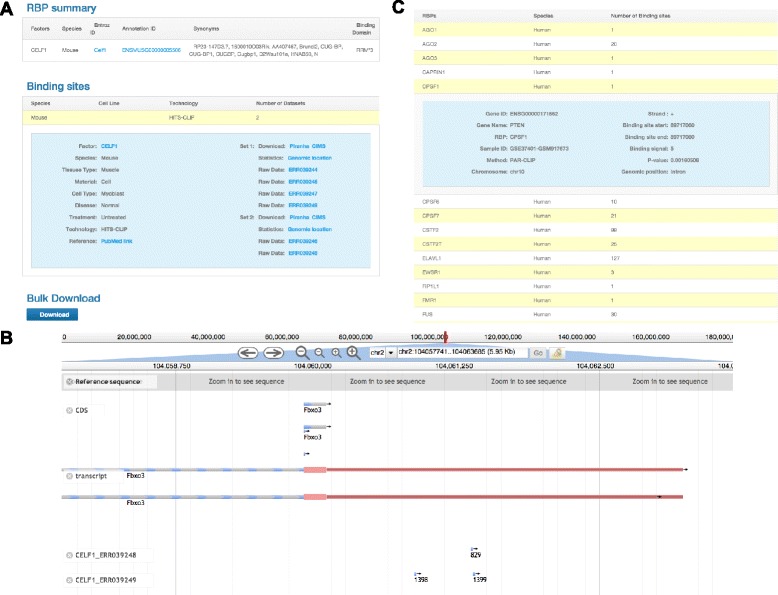
**The utility of CLIPdb. (A)** An example of the “Factor” view for *CELF1*. **(B)** Visualization of *CELF1* binding sites in the untranslated region of the *Fbxo3* gene using the Genome browser. **(C)** An example of searched binding sites for *PTEN*.

As an example, if a user is interested in the *CELF1* binding sites, the user enters or selects “*CELF1*” under the factor view tab. The database returns two CLIP-seq samples (all from mouse) (Figure 3A). For each CLIP-seq sample, the user can link to the raw CLIP-seq data resource in GEO. The database also provides a downloadable BED file containing the binding sites of each CLIP-seq sample. The transcriptome-wide binding sites from each sample are annotated with genomic elements, which are summarized using pie charts. Although the CLIP-seq data actually contain strandness information [5-7], some peak-calling tools (e.g., Piranha) do not provide strandness information for the binding sites. Thus, CLIPdb also provides alternative binding sites identified using other more specialized strand-sensitive peak-calling tools (e.g., CIMS package for HITS-CLIP and iCLIP, and PARalyzer for PAR-CLIP). Additionally, the user may download all the binding sites for each factor (in aggregates of samples) and for each cell line (in aggregates of RBPs).

The user may visualize the transcriptome-wide binding sites with different p-value cutoffs (0.01 or 0.001, assigned by Piranha software [18]) through the “browser” module. The smaller the p-value, the less likely the binding site represents background noise. For example, three binding sites in the UTR region of the *Fbxo3* gene are shown using Jbrowse [30] (Figure 3B). The user may simultaneously select multiple or all CLIP-seq data sets to investigate RBP co-binding patterns in the *Fbxo3* gene.

### Searching binding sites for a given gene

Users often need to know the RBPs that bound the genes of interest profiled by public CLIP-seq data. Alternatively, users may be interested in knowing whether two proteins frequently compete or co-bind for the same site, in which case each of the proteins may be expected to occupy the binding site under different conditions. The binding target search tool enables users to explore the answers to these questions.

As another example, if a user is interested in the mRNA binding sites for human phosphatase and tensin homolog (PTEN), the user enters or selects “*PTEN*” in the search box for humans in the “binding target search” module. The database returns 33 RBPs that have binding sites in the *PTEN* transcript (Figure 3C). The user can obtain detailed information about these binding sites, including the chromosome location, binding strength, p-value, and the CLIP-seq sample and study from which the results were acquired. Importantly, the interactions between RBPs and lncRNAs are not well analyzed in the currently available CLIP-seq studies. With increasing evidence indicating that lncRNAs interact with various RBPs to form complexes [31], we anticipate that the integrated information provided by CLIPdb may lead to novel hypotheses and interesting discoveries.

### Future directions

As the number of publicly available CLIP-seq data sets continues to increase, we will continue to update the database for newly published CLIP-seq studies. As CLIP-seq assays are applied to more RBPs obtained from a broader set of species, cell lines and tissues, data reuse and integrative analysis may become a greater challenge. Thus, we will provide user-friendly CLIP-seq data analysis pipelines for users, including pre-processing, mapping, peak calling and motif identification. In addition to CLIP-seq data, we plan to include ribosome profiling data in the future. Integrating CLIP-seq data and ribosome profiling data will greatly improve our understanding of post-transcriptional regulatory mechanisms. In addition to CLIP-seq experiments, many other low-resolution data are included in other databases (e.g., NPInter [15]). Because these resources provide valuable and more complete information on RBP-RNA interactions, they may also be included in future versions of CLIPdb.

## Conclusions

CLIPdb is a more complete and better-curated database focused on CLIP-seq data sets than those currently available, providing genome-wide, high-resolution binding sites. Characterizing the post-transcriptional regulatory networks mediated by RNA-binding proteins is a problem of great interest. CLIP-seq technologies (i.e., HITS-CLIP, PAR-CLIP and iCLIP) have proven their power in deciphering complex codes at the single nucleotide level. Here, we presented a useful resource for the reuse and mining of publicly available CLIP-seq data. To our knowledge, CLIPdb is the most comprehensive database available for use in examining published CLIP-seq studies. It provides high-resolution RBP binding sites across whole genomes, including both mRNA (CDSs plus UTRs) and non-coding RNA.

To make the binding sites comparable and the data integrative across multiple samples from different batches, we used uniform pre-processing and peak-calling procedures to identify RBP binding sites from raw CLIP-seq data. CLIPdb embraces all variations of CLIP technologies, including PAR-CLIP with its T-to-C conversions, HITS-CLIP with its deletions at the crosslinked sites, and iCLIP with its truncations at the crosslinked sites. We systematically identified binding sites using Piranha, a tool that has demonstrated success across three CLIP-seq variants [18]. Thus, CLIPdb uniformly scores or ranks binding sites from various CLIP-seq experiments, which is important for the integrative analysis of these published CLIP-seq data. However, Piranha was not optimized for specific CLIP-seq technology, such as PAR-CLIP for which T-to-C conversions indicate binding events. Thus, we also provided binding sites identified by specialized peak-calling tools for specific CLIP-seq technology. In addition, the various sequencing depths of the collected CLIP-seq data sets may significantly affect peak calling. We found very few (<10) binding sites generated from some CLIP-seq data sets because of low sequencing quality and depth (usually less than 1 M unique mapped reads). We do not include these data sets in CLIPdb because the mapping size would have significant impact on the binding sites being called. Users should exercise caution while optimizing the peak-calling procedure when reusing these published CLIP-seq data.

Re-analysis of publicly available CLIP-seq data may generate novel hypotheses. For example, we found that more than 10 orthologous RBPs have been studied using CLIP-seq technologies. Our data will be useful for comparative analyses of their binding sites and may uncover evolutionary signatures for RBPs binding. In addition, CLIPdb provides a unified data set annotation vocabulary as well as several queries and views of the data as a convenience for users. Therefore, CLIPdb should provide additional insights into RNA-protein interaction networks, such as lncRNA-protein interactions and their cellular functions.

## Availability and requirements

CLIPdb is freely accessible at http://clipdb.ncrnalab.org. All data are available for download from the database. The programming languages include PHP, MySQL, HTML and JavaScript. A license is not required and there are no restrictions for use by non-academics.

## Additional files

Additional file 1:
**Summary of CLIP-seq data sets we used and processed.**
Additional file 2:
**Summary of the functional groups of RBPs in human, mouse, worm and yeast.**
Additional file 3:
**Genomic distributions of RBP binding sites identified by Piranha (Figure S1, in worm and yeast) and specialized tools (Figure S2).**
