# Supplementary material for: CLIPdb: a CLIP-seq database for protein-RNA interactions
Source: BMC Genomics. 2015 Feb 5;16(1):51. doi: 10.1186/s12864-015-1273-2 (PMC4326514; doi:10.1186/s12864-015-1273-2)
Supplement: Additional file 3: — Genomic distributions of RBP binding sites identified by Piranha (Figure S1, in worm and yeast) and specialized tools (Figure S2). [file 12864_2015_1273_MOESM3_ESM.pdf]

Supplementary Materials

CLIPdb: a CLIP-seq database for protein-RNA interactions

Yu-Cheng T. Yang, Chao Di, Boqin Hu, Meifeng Zhou, Yifang Liu, Nanxi Song, Yang Li, Jumpei Umetsu, and Zhi John Lu

Supplementary Figure 1

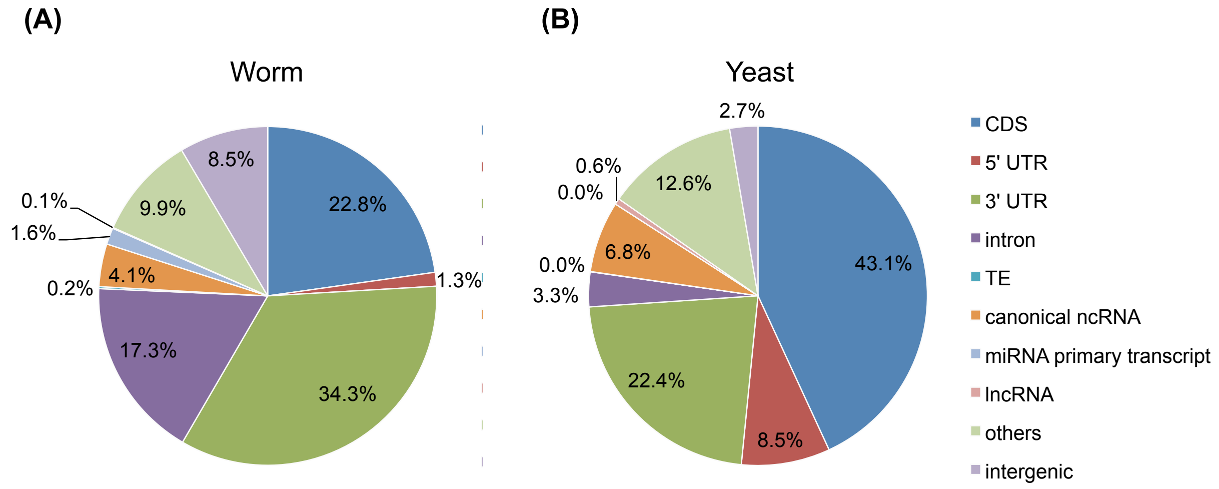

Genomic distributions of RBP binding sites identified by Piranha in worm (A) and yeast (B).

Supplementary Figure 2

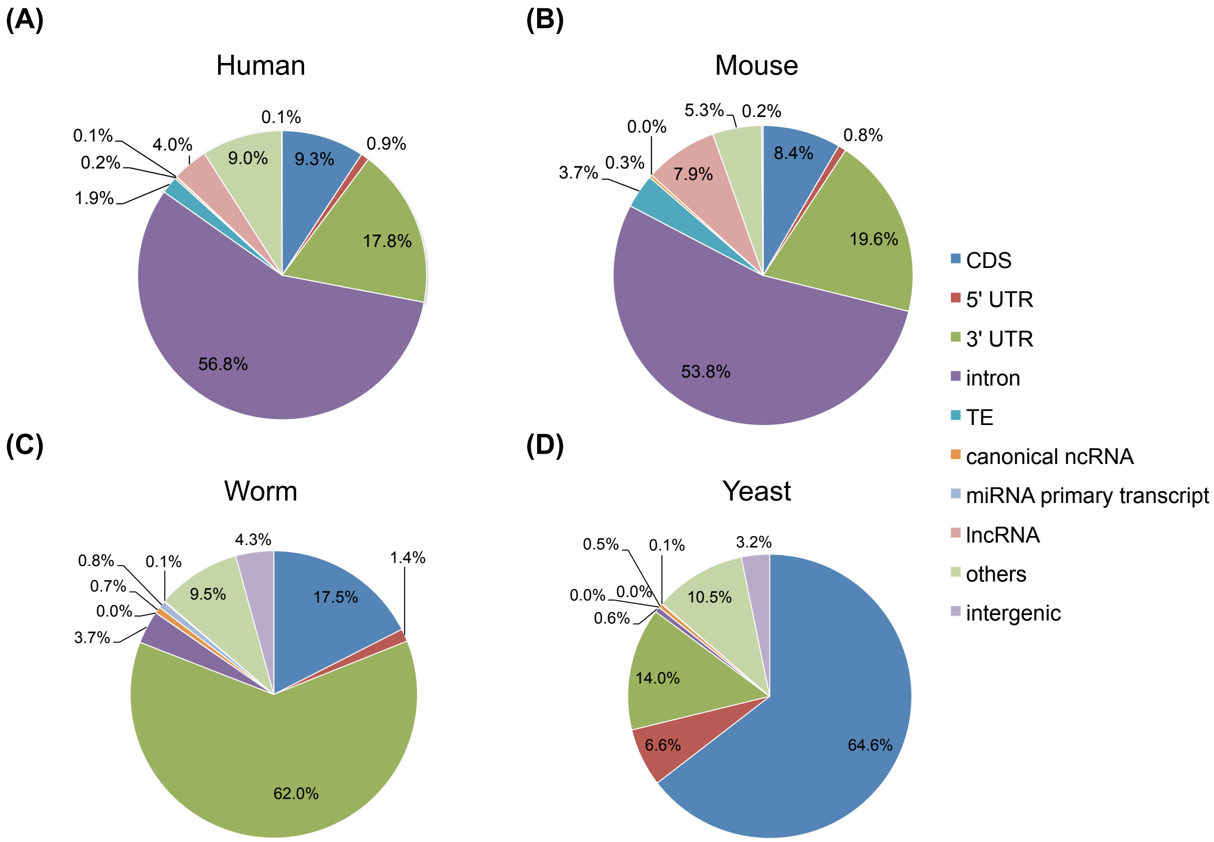

Genomic distributions of RBP binding sites identified by specialized tools in human (A), mouse (B), worm (C), and yeast (D). The genomic distributions of RBP binding sites generated by different methods are compared. In general, for large data sets (i.e., human and mouse), the genomic distribution of peaks called by different methods is stable. The specialized methods are more sensitive and capable of finding more peaks than the unified method (i.e., Piranha) [1].

Supplementary File 1

Summary of the CLIP-seq data sets we used and processed.

Supplementary File 2

Summary of the functional groups of RBPs in human, mouse, worm and yeast.

References

1. Wang T, Xie Y, Xiao G: **dCLIP: a computational approach for comparative CLIP-seq analyses.** *Genome biology* 2014, **15**(1):R11.
